# Disorder-driven non-Anderson transition in a Weyl semimetal

**DOI:** 10.1073/pnas.2508569122

**Published:** 2025-10-09

**Authors:** Cong Li, Yang Wang, Jianfeng Zhang, Hongxiong Liu, Wanyu Chen, Guowei Liu, Hanbin Deng, Timur K. Kim, Craig Polley, Balasubramanian Thiagarajan, Jiaxin Yin, Youguo Shi, Tao Xiang, Oscar Tjernberg

**Affiliations:** ^a^Department of Applied Physics, Kungliga Tekniska högskolan Royal Institute of Technology, Stockholm 11419, Sweden; ^b^Beijing National Laboratory for Condensed Matter Physics, Institute of Physics, Chinese Academy of Sciences, Beijing 100190, China; ^c^Department of Physics, Southern University of Science and Technology, Shenzhen, Guangdong 518055, China; ^d^Diamond Light Source, Harwell Campus, Didcot OX11 0DE, United Kingdom; ^e^MAX IV Laboratory, Lund University, Lund 22100, Sweden

**Keywords:** non-Anderson transition, ARPES, Weyl semimetal, electronic structure

## Abstract

For several decades, it was widely believed that a noninteracting disordered electronic system could only undergo an Anderson metal–insulator transition due to Anderson localization. However, numerous recent theoretical works have predicted the existence of a disorder-driven non-Anderson phase transition that differs from Anderson localization. The frustration lies in the fact that this non-Anderson disorder-driven transition has not yet been experimentally demonstrated in any system. Here, using angle-resolved photoemission spectroscopy, we present a case study of observing the non-Anderson disorder-driven transition by visualizing the electronic structure of the Weyl semimetal NdAlSi on surfaces with varying amounts of disorder. Our observations reveal that strong disorder can effectively suppress all surface states in the Weyl semimetal NdAlSi, including the topological surface Fermi arcs. This disappearance of surface Fermi arcs is associated with the vanishing of the topological invariant, indicating a quantum phase transition from a Weyl semimetal to a diffusive metal. These observations provide direct experimental evidence of the non-Anderson disorder-driven transition occurring in real quantum systems, a finding long anticipated by theoretical physicists.

## Significance

Metal–insulator transitions are traditionally attributed to Anderson localization, where disorder traps electrons. However, recent theories predict a new type of transition–non-Anderson disorder-driven transitions–that occur without localization. This study provides direct experimental evidence of such a transition. Using angle-resolved photoemission spectroscopy, we show that disorder in the Weyl semimetal NdAlSi causes the complete disappearance of its topological surface Fermi arcs, without signs of localization. This indicates a transition from a topological semimetal to a diffusive metallic phase. Our results bridge a long-standing gap between theory and experiment, revealing a fundamentally different way in which disorder reshapes quantum materials.

Disorder-driven transitions are a longstanding area of interest in the field of condensed matter physics. For a long time, it was generally believed that the Anderson localization (1) transition is the only possible disorder-driven transition in noninteracting systems. Recently, however, extensive theoretical works have proposed that a broad class of systems may exhibit a new kind of disorder-driven transitions occurring prior to Anderson localization, which are manifested by the critical behavior of the disorder-averaged density of states and other observable phenomena (2–34). The theory suggests that such non-Anderson disorder-driven transition is a general phenomenon that occurs in all quantum systems in sufficiently high dimensions (12, 13, 28), attracting significant attention, especially in the study of three-dimensional (3D) topological semimetals (7–34).

Topological semimetals are a class of materials that possess unique electronic properties due to their topological characteristics, including Dirac semimetals, Weyl semimetals, and nodal line semimetals (31, 35, 36). Recently, extensive theoretical studies have pointed out that Weyl semimetals are a potential platform for observing the non-Anderson disorder-driven transition (7–34). In Weyl semimetals, the topological surface Fermi arc (SFA) can survive weak disorder (24, 26). However, as the disorder increases beyond a certain threshold, the SFA will disappear (24, 26), accompanied by a non-Anderson disorder-driven quantum phase transition from the Weyl semimetal to a diffusive metal state (14, 18, 24, 27, 34). However, so far, no experiments have observed the non-Anderson transition where disorder causes the disappearance of SFA in Weyl semimetals. On the contrary, experiments have demonstrated that SFA remains robust even in the presence of disorder (37, 38). In fact, over the past decade, the existence of non-Anderson disorder-driven quantum phase transitions also has been predicted in various quantum systems beyond topological semimetals, including 1D and 2D arrays of ultracold trapped ions, chiral superconductors and quantum kicked rotors, and numerous new theoretical predictions continue to emerge (2–34). However, experimental evidence for the non-Anderson transition has remained elusive, fueling speculation that it may be nothing more than a theoretical construct or an abstract mathematical model devised by theoretical physicists.

In this paper, employing angle-resolved photoemission spectroscopy (ARPES) and scanning tunneling microscope (STM) measurements as well as density functional theory (DFT) calculations, we systematically investigate the near surface electronic structure of the magnetic Weyl semimetal NdAlSi (39–41), on surfaces with varying amount of disorder. Our findings reveal that all surface states, including the topological surface Fermi arcs (SFAs), are completely suppressed on the strong disordered surface of NdAlSi. To experimentally simulate increasing disorder, we conducted time-dependent electronic structure measurements on a highly ordered surface of NdAlSi. The results demonstrate that the SFAs can survive weak disorder, but the shape of the SFAs change. In strong disorder, the SFAs completely dissolve into the bulk metallic background, indicating the emergence of the non-Anderson disorder-driven quantum phase transition from a Weyl semimetal to a diffusive metal in a quasi-two-dimensional system within the photoelectron probing depth. Our findings provide direct experimental evidence for the existence of the non-Anderson disorder-driven transition in quantum systems, transcending purely mathematical concepts.

**Fig. 1.**
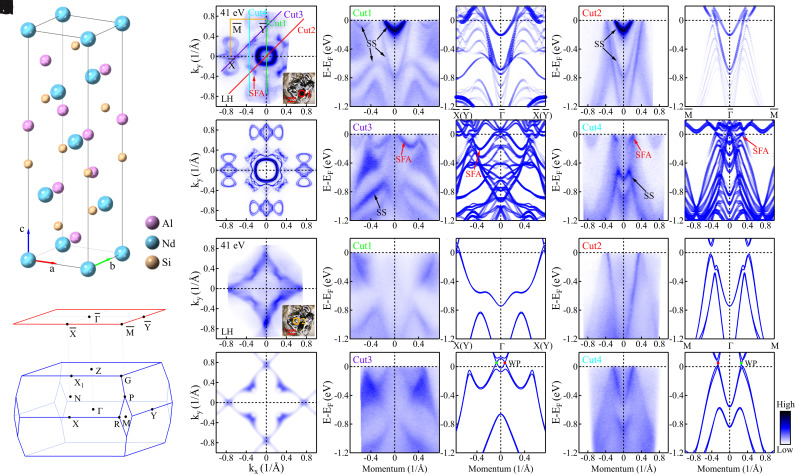
Surface and bulk electronic structures of NdAlSi. (*A*) The crystal structure of NdAlSi with the space group $I4_{1}md$ (no. 109). (*B*) The 3D BZ of the original unit cell of NdAlSi, and the corresponding two-dimensional BZ projected on the (001) plane (red lines) in the pristine phase in (*A*). (*C*) Fermi surface of NdAlSi measured with photon energy of 41 eV under LH polarization in the area 1 (red circle) of sample. (*D*) Surface projected DFT calculated Fermi surface on the terminal surface of Nd atoms cleavage at the Al-Nd layer with considering two domain structures. (*E*) Measured band dispersion along $\bar{Y}-\bar{\Gamma }-\bar{Y}$ (Cut1) direction in the area 1 of sample under LH polarization. (*F*) The corresponding surface projected DFT calculations of the bands along $\bar{Y}-\bar{\Gamma }-\bar{Y}$ direction, considering the two domain structures. (*G*, *H*) The similar as (*E*, *F*) but along $\bar{M}-\bar{\Gamma }-\bar{M}$ (Cut2) direction. (*I*, *J*) The similar as (*E*–*F*) but along Cut3 direction. (*K*, *L*) The similar as (*E*, *F*) but along Cut4 direction. While Cut3 and Cut4 go right through the Weyl points. (*M*) Fermi surface of NdAlSi measured with photon energy of 41 eV under LH polarization in the area 2 (orange circle) of sample. According to the previous report, bulk state measurements with photon energy of 41 eV corresponds to the k_*z*_ ∼ 0 π/c plane (40). (*N*) The DFT calculated bulk Fermi surface at the k*_z_* = 0 *π*/*c* plane which integrate 0±0.1 π/c of BZ along k_*z*_ direction. (*O*) The similar measurements as (*E*) but measured in the area 2 of sample. (*P*) The DFT calculated bulk band structures along Y−Γ−Y (Cut1) direction. (*Q*, *R*) The similar as (*O*, *P*) but along $\bar{M}-\bar{\Gamma }-\bar{M}$ (Cut2) direction. (*S*, *T*) The similar as (*O*, *P*) but along Cut3 direction. (*U*, *V*) The similar as (*O*, *P*) but along Cut4 direction. The trivial surface states (SSs) are marked by black arrows, and the SFAs are marked by red arrows which was well studied in ref. (40). The green and red dots in (*T*) and (*V*) mark the Weyl points (WPs), while the red dots representing nodes with chiralities +1 and green dots representing −1.

To achieve a comprehensive understanding of the electronic structure of NdAlSi, we first performed DFT calculations on it (see *SI Appendix*, section 1 for details). In conjunction with DFT calculations, we performed ARPES measurements on NdAlSi. The NdAlSi crystal after cleavage shows flat areas (area 1 in Fig. 1*C*) as well as areas with multiple steps (area 2 in Fig. 1*M*). The corresponding location can be found by spatial scanning of the sample. When the measurement position is in area 1 (red circle in Fig. 1*C*), the measured electronic structures are shown in Fig. 1*C*, *E*, *G*, *I*, and *K*. It is seen that the measured electronic structures are in good agreement with the surface projected DFT Fermi surface calculations for the Nd terminated surface at the Al-Nd layer, taking into account the two domain structures (Fig. 1 *D*, *F*, *H*, *J*, and *L*) visible due to the fact that the light spot covers two orthogonal domains simultaneously (40). However, when the measurement position is in area 2 (orange circle in Fig. 1*M*), the measured electronic structures (Fig. 1*M*, *O*, *Q*, *S*, and *U*) are entirely different from those measured in area 1. First, we rule out the possibility that the electronic structure measured on the uneven surface (area 2) comes from some other surface termination by comparing to surface projected DFT calculations on all possible surface terminations (see *SI Appendix*, section 2 for details). To further clarify, DFT bulk band structure calculations were carried out along X(Y)−Γ−X(Y) (Cut1, Fig. 1*P*), M−Γ−M (Cut2, Fig. 1*R*), Cut3 (Fig. 1*T*), and Cut4 (Fig. 1*V*). We find that all the band features in the measured band dispersions (Fig. 1 *O*, *Q*, *S*, and *U*) are captured by the corresponding bulk band structure calculations (Fig. 1 *P*, *R*, *T*, and *V*). Furthermore, DFT calculations for the bulk Fermi surface at the k_*z*_ = 0 π/c plane, integrating 0±0.1 π/c of the BZ along the k_*z*_ direction, are shown in Fig. 1*N*. The calculated bulk Fermi surface (Fig. 1*N*) is also in good agreement with the Fermi surface measured in area 2 with a photon energy of 41 eV (Fig. 1*M*).

It appears clear that the electronic structure measured in area 1 is mainly from the surface states and the electronic structure measured in area 2 is mainly from the bulk states. This provides us with an opportunity to directly study the bulk electronic structure of NdAlSi with high energy and momentum resolution. Furthermore, it is worth noting that in addition to the trivial surface states, the SFAs measured on the ordered surface also completely disappear on the disordered surface (Fig. 1*S* and *U*). In addition, the bulk states measured on the disordered surface do not show any obvious degradation or broadening as compared with the flat surface, some bands even look sharper (see *SI Appendix*, section 3 for details). We note that in the context of a three-dimensional (3D) Weyl semimetal, the bulk-boundary correspondence highlights that Weyl points are always associated with SFAs. The disappearance of SFAs connecting the projected Weyl points in area 2 seem to challenge the bulk-boundary correspondence.

**Fig. 2.**
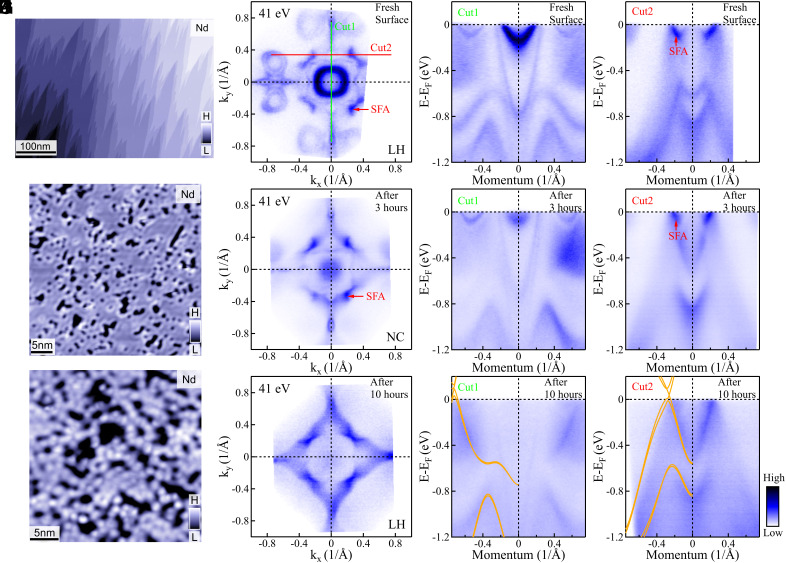
The STM measurements and evolution of the electronic structure with time. (*A*) STM images of NdAlSi measured at 4.7 K on Nd atom terminated surface cleavage at the Al-Nd layer. (*A*) is the overview image. (*B* and *C*) are the magnified topography images measured at different positions. Scan conditions: (*A*) 1 V, 0.1 nA, (*B*) 0.5 V, 0.5 nA, and (*C*) 0.01 V, 0.1 nA. (*D*–*G*) Fermi surface of NdAlSi measured with photon energy of 41 eV under LH (*D*, *F*, and *G*) and NC (*E*) polarizations on flat area of the fresh sample (*D*), the sample after 3 h of continuous measurement (*E*), the sample after 10 h of continuous measurement (*F*). (*G*–*I*) The corresponding band dispersions along $\bar{Y}-\bar{\Gamma }-\bar{Y}$ [Cut1, green line in (*D*)] directions. (*J*–*L*) The corresponding bands along Cut2 [red line in (*D*)] directions. The corresponding DFT bulk calculations are overlaid on (*I* and *L*). The red arrows mark the SFA.

In order to understand the mechanism behind this, we carried out STM measurements on an uneven area as shown in Fig. 2 *A*–*C*. (STM measurements on a flat area are presented in *SI Appendix*, section 4). Fig. 2*A* shows the overview STM image of the Nd terminated surface which exhibits multiple steps. In this area, we choose two different positions to do magnified topography image scans, as shown in Fig. 2 *B* and *C*. Considerable disorder is observed in the form of atomic clusters and holes. In theoretical simulations (24, 26), it has been found that although the SFAs in Weyl semimetals are topologically protected, they are not robust against strong disorder. While the SFAs can survive in weak disorder, they dissolve into the bulk metallic background as the disorder increases (24, 26). In strong disorder, the disappearance of the SFAs is directly related to the vanishing of the bulk topological invariant. Therefore, the disappearance of the SFA is accompanied by a Weyl semimetal-diffusive metal quantum phase transition. The metallic phase is also supporting quasi-particles with a finite lifetime and mean free path but is topologically trivial. Thus, the disappearance of the SFAs does not invalidate the bulk-boundary correspondence (24).

To study the electronic structure evolution with increasing disorder, we carried out time-dependent electronic structure measurements in the ordered areas of the sample. In this case, all of the surface states (trivial surface states and SFAs) can be clearly observed when measured on the ordered and freshly cleaved sample, as shown in Fig. 2 *D*, *G*, and *J*. However, three hours later, the surface states appear blurred (Fig. 2 *E*, *H*, and *K*) relative to the newly cleaved sample, and some structures of the surface state even shift to higher binding energy. Ten hours later, all of the surface states, including SFAs, seem to be completely suppressed, leaving only the bulk states, as shown in Fig. 2 *F*, *I*, and *L*. This is similar to the electronic structure measured on the disordered surface of the newly cleaved sample (Fig. 1 *M*, *O*, and *U*). A reasonable explanation for the evolution of the above electronic structure over time is that the impurities are adsorbed at low temperatures under ultraviolet (UV) irradiation (42, 43), which leads to surface disorder on the sample, thus affecting the near surface electronic structure of the sample. During the same time, the SFAs also evolve. In the beginning, the SFAs appear very sharp (Fig. 2*J*), and after 3 h of continuous measurement, they are gradually smoothed out (Fig. 2*K*) until they disappear completely after 10 h of continuous measurement (Fig. 2*L*) (The more detailed energy distribution curve analysis see *SI Appendix*, section 6). At the same time, the diamond-shaped Fermi surface (marked by green arrows in Fig. 2 *E* and *F*) gradually appears and eventually dominate as shown in Fig. 2*F*. In addition, we also performed surface potassium deposition on NdAlSi to simulate the process of increasing surface disorder. During this process, we observed a similar trend: As the disorder introduced by potassium deposition increased, the SFA was gradually suppressed and eventually disappeared completely (see *SI Appendix*, section 7 for details).

**Fig. 3.**
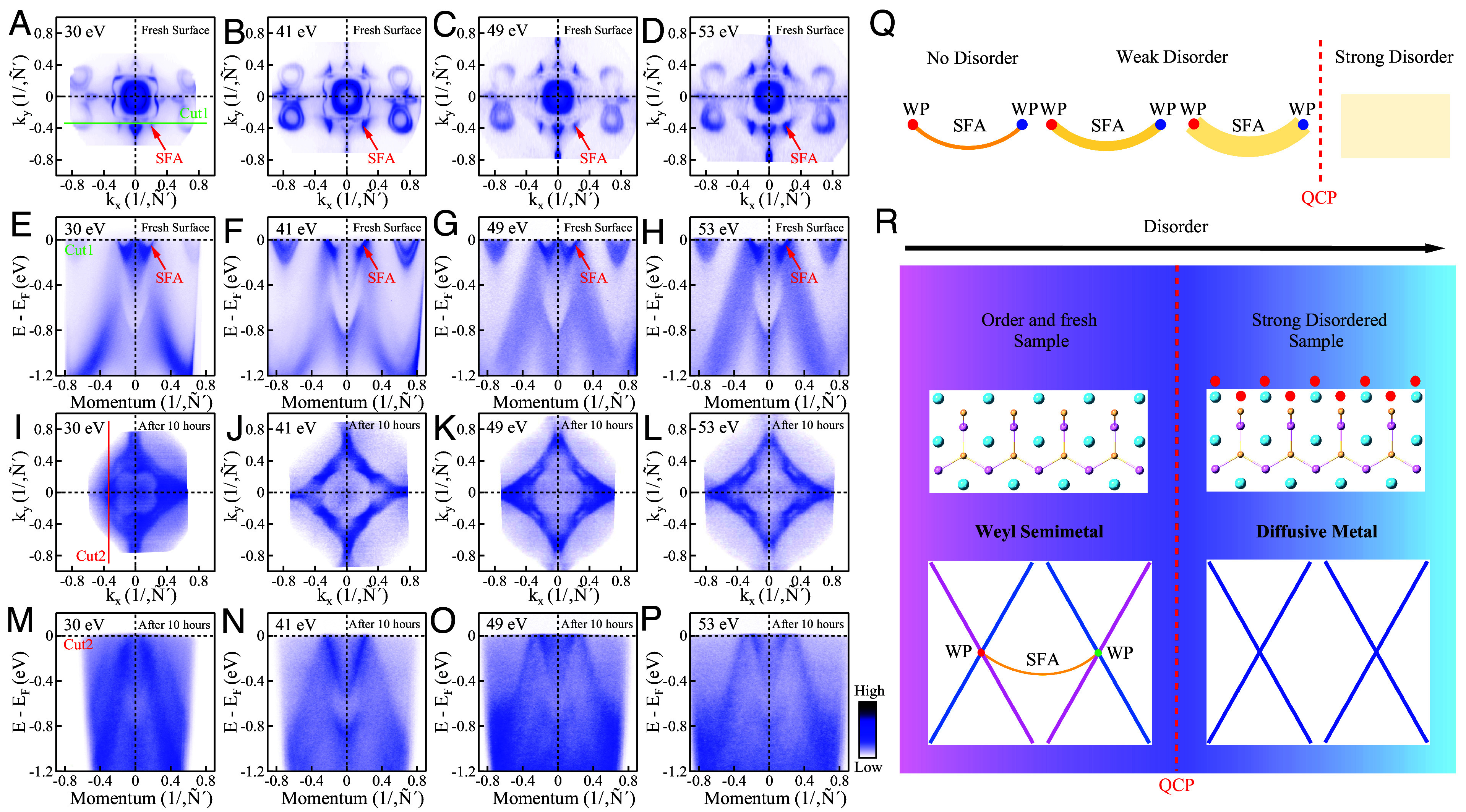
Weyl semimetal-diffusive metal quantum phase transition. (*A*–*D*) Photon energy–dependent Fermi surface measured on fresh surface of flat sample with photon energies of 30 eV (*A*), 41 eV (*B*), 49 eV (*C*), and 53 eV (*D*). (*E*–*H*) Photon energy–dependent band structures measured on fresh surface of flat sample with photon energies of 30 eV (*E*), 41 eV (*F*), 49 eV (*G*), and 53 eV (*H*) along Cut1 in (*A*). The red arrows mark the SFAs. (*I*–*P*) The similar measurements as (*A*–*K*) but measured on the surface after 10 h of continuous measurement. (*Q*) Schematic diagram of SFA suppressed by disorder. (*R*) Schematic phase diagram of the Weyl semimetal as the increase of disorder. The red dashed lines mark the quantum critical point (QCP).

In order to further study and disentangle the bulk vs. surface electronic structure in NdAlSi, we performed photon energy-dependent Fermi surface measurements, as shown in Fig. 3 *A*–*P*. Fig. 3 *A*–*D* show the photon energy–dependent Fermi surfaces measured on the fresh and ordered surface with photon energies of 30 eV (Fig. 3*A*), 41 eV (Fig. 3*B*), 49 eV (Fig. 3*C*), and 53 eV (Fig. 3*D*). The corresponding photon energy–dependent band structures along Cut1 (green line in Fig. 3*A*) are shown in Fig. 3 *E*–*H*. The SFAs are clearly observed at all photon energies (marked by red arrows in Fig. 3 *A*–*H*). After 10 h of continuous measurement, as the disorder increases, the SFAs completely dissolve into the bulk metallic background. At this point the SFAs completely disappear from the photon energy-dependent measurements (Fig. 3 *I*–*P*). The process of SFA dissolution is schematically shown in Fig. 3*Q* (24). At first, the SFAs show very sharp features (Figs. 2 *D* and *J* and 3*A*–*H*). In weak disorder, the SFAs still survive (Fig. 2 *E* and *K*), but become less well defined as the disorder increases. When the disorder increases sufficiently, the SFAs completely disappear. At the same time, the diamond-shaped Fermi surface appears (Fig. 3 *I*–*L*). According to the previous report (40), bulk state measurements with photon energy of 41 eV corresponds to k_*z*_ ∼ 0 π/c plane, the photon energy of 30 eV and 53 eV corresponds to kz ∼ 1 π/c plane and the photon energy of 49 eV corresponds to k_z_ ∼ 0.67 *π*/c. The observed diamond-shaped Fermi surface is difficult to attribute to the bulk state dispersion, since it exhibit negligible photon energy dependence (see *SI Appendix*, section 9 for details).

Based on the above observations, we propose a conjecture for the underlying process. At first, on the clean ordered surface, the electronic structure is that of a Weyl semimetal. As the disorder increases at the surface, multiple boundaries are created that carry chiral edge states. For weak disorder, a larger number of chiral edge states can be accommodated that smooths out the SFAs. When the surface disorder increases sufficiently, the underlying second ordered layer becomes the topmost ordered layer. This layer does not have full direct contact with the vacuum and lacks the out-of-plane translational symmetry of the previous bulk state. Consequently, the associated two-dimensional electronic structure associated with this layer, characterized by the diamond-shaped Fermi surface, retains some aspects of the electronic structure of the bulk state. This view points to the measured electronic structures (Fig. 3 *I*–*P*) originating from the underlying second layer. At this stage, the SFA is effectively relocated to an ordered interface layer beneath a disordered surface layer. As a result, the bulk topological invariants of this buried ordered layer vanish, indicating that it no longer exhibits the characteristics of a Weyl semimetal. With continued adsorption, the underlying bulk termination layer becomes increasingly separated from the vacuum, until the ordered layer exhibiting the SFA lies beyond the photoelectron probing depth.

Owing to the surface sensitivity of ARPES, our claim of a non-Anderson disorder-driven quantum phase transition from a Weyl semimetal to a diffusive metal–a metallic state where electronic transport is governed by diffusive electron scattering–is confined to a quasi-two-dimensional region within the photoelectron probing depth. This transition is realized by gradually introducing sufficient disorder–via surface adsorption or surface disruption–into a region spanning a few unit cells beneath the surface. However, this does not imply that regions beyond the ARPES probing depth are unaffected by surface disorder. Rather, our observations reveal the fragility of the topological surface response to disorder and highlight a previously unexplored regime of disorder-induced topological evolution. The key to demonstrating the existence of a disorder-driven non-Anderson topological quantum phase transition lies in finding evidence that the topological surface states disappear prior to other band features. Prior to this, the disappearance of disorder-induced topological surface states had also been observed in Bi_2_Se_3_ (44, 45). However, it was not recognized at the time that this phenomenon could, to some extent, be identified as a disorder-driven non-Anderson quantum phase transition, since the topological surface states vanish before the bulk states as disorder increases. However, discussions of disorder-driven non-Anderson topological quantum phase transitions often consciously avoid topological insulator systems. The main reason is that, strictly speaking, in a topological insulator, the bulk is insulating while the surface states are metallic. When the surface states of a topological insulator are completely suppressed by disorder, the system becomes a true insulator, and the corresponding transition is an Anderson localization–driven transition from a topological insulator to an ordinary insulator. However, in many cases, topological insulators can have bulk states crossing the Fermi level due to doping. If, when the topological surface states are fully suppressed, bulk states still cross the Fermi level, then to some extent this can also be regarded as a disorder-driven non-Anderson topological quantum phase transition occurring in the material.

In summary, by carrying out ARPES measurements on the Weyl semimetal NdAlSi, we found that all of the surface states, trivial surface states as well as SFAs, were suppressed on strongly disordered surfaces. The temporal evolution of the electronic structure observed on originally clean and ordered surfaces provides an experimental way of following increasing disorder in this Weyl semimetal. It was found that the SFAs can survive in weak disorder but as the disorder increases, the SFAs dissolve into the bulk metallic background until they disappear completely. The disappearance of the SFAs are related to the vanishing of the topological invariant, indicating a Weyl semimetal-diffusive metal quantum phase transition. Our results provide direct evidence of non-Anderson disorder-driven transitions in Weyl semimetal NdAlSi, demonstrating that this transition is no longer merely a theoretical model. This finding also opens broad avenues for the study of new quantum states in topological materials caused by non-Anderson transitions.

## Materials and Methods

### Sample.

Single crystals of NdAlSi were grown from Al as flux. Nd, Al, Si elements were sealed in an alumina crucible with the molar ratio of 1:10:1. The crucible was finally sealed in a highly evacuated quartz tube. The tube was heated up to 1,273 K, maintained for 12 h and then cooled down to 973 K at a rate of 3 K per hour. Single crystals were separated from the flux by centrifuging. The Al flux attached to the single crystals were removed by dilute NaOH solution.

### ARPES Measurements.

High-resolution ARPES measurements were performed at the I05 beamline of the Diamond synchrotron and at the Bloch beamline of MAX IV synchrotron. The total energy resolution (analyzer and beamline) was set at 15 ∼ 20 meV for the measurements. The angular resolution of the analyzer was ∼0.1°. The beamline spot size on the sample was about 70 μm × 70 μm at the I05 beamline of the Diamond synchrotron and about 10 μm × 12 μm at the Bloch beamline of the MAX IV synchrotron. The samples were cleaved in situ and measured in ultrahigh vacuum with a base pressure better than 1.0 × 10^−10^ mbar at about 10 K at the I05 beamline of the Diamond synchrotron and about 18 K at the Bloch beamline of the MAX IV synchrotron.

### STM Measurements.

STM experiments were conducted using an ultrahigh vacuum low-temperature STM (Unisoku, USM1300) under a base pressure below 1 × 10^−10^ mbar. High-quality NdAlSi single crystals, measuring up to 3 mm× 3 mm × 3 mm, were bisected prior to being affixed to beryllium copper sheets. The crystal was mechanically cleaved in situ at 78 K and promptly placed into the microscope head, which was already at the base temperature of He4 (4.7 K). Topographic images were obtained using Ir/Pt tips in constant-current mode at 4.7 K.

### DFT Calculations.

The electronic structure calculations for NdAlSi were performed based on the DFT (46, 47) as implemented in the VASP package (48, 49). The generalized gradient approximation of Perdew–Burke–Ernzerhof type (50) was chosen for the exchange-correlation functional. The projector augmented wave method (51, 52) was adopted to describe the interactions between valence electrons and nuclei. In calculation, the Nd pseudopotential was chosen without the 4f electrons. The kinetic energy cutoff of the plane-wave basis was set to be 350 eV. A 16 × 16 × 16 Monkhorst–Pack grids (53) was used for the BZ sampling. For describing the Fermi–Dirac distribution function, a Gaussian smearing of 0.05 eV was used. When studying the surface states of NdAlSi, we employed a 36 atomic layer slab system with 20 Å vacuum layer.

## Supplementary Material

Appendix 01 (PDF)

## Data Availability

All study data are included in the article and/or *SI Appendix*.
