# Supplementary material for: Disorder-driven non-Anderson transition in a Weyl semimetal
Source: Proc Natl Acad Sci U S A. 2025 Oct 9;122(41):e2508569122. doi: 10.1073/pnas.2508569122 (PMC12541400; doi:10.1073/pnas.2508569122)
Supplement: Supplementary file 1 — Appendix 01 (PDF) [file pnas.2508569122.sapp.pdf]

Supplementary Material for

Disorder Driven Non-Anderson Transition in a Weyl Semimetal

Cong Li[1,*], Yang Wang[1], Jianfeng Zhang[2], Hongxiong Liu[2], Wanyu Chen[1], Guowei Liu[3], Hanbin Deng[3], Timur K. Kim[4], Craig Polley[5], Balasubramanian Thiagarajan[5], Jiaxin Yin[3], Youguo Shi[2], Tao Xiang[2], Oscar Tjernberg[1,*]

[1]*Department of Applied Physics, KTH Royal Institute of Technology, Stockholm 11419, Sweden*

[2]*Beijing National Laboratory for Condensed Matter Physics, Institute of Physics, Chinese Academy of Sciences, Beijing 100190, China*

[3]*Department of Physics, Southern University of Science and Technology, Shenzhen, Guangdong 518055, China*

[4]*Diamond Light Source, Harwell Campus, Didcot, OX11 0DE, United Kingdom*

[5]*MAX IV Laboratory, Lund University, 22100 Lund, Sweden*

[*]*Corresponding authors: conli@kth.se, oscar@kth.se*

1. Crystal structure and calculated electronic structure of NdAlSi

To achieve a comprehensive understanding of the electronic structure of NdAlSi, we first performed DFT calculations on it, as shown in Fig. S1. Fig. S1a shows the crystal structure of NdAlSi, which crystallizes in the tetragonal structure with the space group $I4_1md$ (no. 109)[1]. The corresponding three-dimensional (3D) Brillouin zone (BZ) of NdAlSi is shown in Fig. S1b, which defines the high symmetry points in the BZ. According to previous reports, the Al-Nd layer is the dominating cleavage plane in NdAlSi[2, 3]. DFT calculations predict that the Nd terminated surface from a cleave at the Al-Nd layer forms a complex surface state (Fig. S1c and S1f) which differs from the electronic structure of the bulk state (Fig. S1d and S1g)[2]. Furthermore, DFT calculations of the bulk states, integrated along the k_z direction as shown in Fig. S1e and S1h, as well as the surface projected DFT calculations (Fig. S1c and S1f), provide an overview of the bulk and surface electronic structure of NdAlSi.

2. Terminal surface dependent Fermi surface calculations of NdAlSi

From the perspective of crystal structure of NdAlSi (Fig. S1a), there are three possible cleavage planes in NdAlSi. These are cleavage at the Si-Al layer, cleavage at the Al-Nd layer and cleavage at the Nd-Si layer. Therefore, there are potentially six different terminal surfaces in NdAlSi corresponding to six different surface electronic structures. To visualize the difference between the six terminal surfaces, we carried out surface projected DFT Fermi surface calculations on these six terminal surfaces are shown in Fig. S2. From the calculations, it can be noted that the electronic structure of different terminal surfaces are expected to differ substantially.

3. Comparison of electronic structures of flat and uneven surfaces

Figures S3a and S3b present the Fermi surfaces measured on the flat and uneven fresh surfaces, respectively. The corresponding X-ray photoelectron spectroscopy (XPS) results are shown in Fig. S3c (flat surface) and S3d (uneven surface). In the uneven (disordered) region, defects in the Nd surface layer above the Si atoms may alter the local chemical environment of Si, leading to slight splitting or shifts in the Si 2p peak (Fig. S3d), as well as a reduction in the spectral intensity of the $2p_{1/2}$ component (Fig. S3c). In contrast, the Al atoms, which lie beneath the Si layer, are less affected. Consequently, no significant

splitting or peak shift is observed in the Al 2p spectrum, although a slight decrease in the $2p_{1/2}$ intensity is present (Fig. S3d). It is worth noting that within the measured energy range, no additional impurity peaks were observed on the freshly cleaved uneven surface, suggesting that the disorder on the fresh uneven surface is likely primarily caused by Nd atom defects.

To compare the electronic structure measured on a flat and uneven surfaces in more detail, we carried out constant energy contour measurements, as shown in Fig. S4a-S4b. Comparing the difference between them can help distinguish features of surface and bulk states. It can be found that although the electronic structure measured on the flat surface is dominated by the surface states, the bulk features can still be found at some energies. Fig. S4c-S4d show the band structures measured along Cut1-Cut7 (red lines in Fig. S4a) which covers half of the BZ. From the position of the Cuts in Fig. S4a, it can be seen that Cut1, Cut2, Cut4 and Cut5 pass right through or near the position of the surface Fermi arc (SFA). Therefore, the SFA projections can be seen in the band measurements of the corresponding Cut in Fig. S4c (marked by red arrows). Compared to the corresponding bands measured in area 2 (Fig. S4d), the SFAs completely disappear. In addition, the bulk states measured on the uneven surface do not show obvious degradation compared with the flat surface (Fig. S4c-S4d), and some bands even look sharper (marked by green arrows in Fig. S4b and S4d).

4. Scanning tunneling microscope measurements of NdASi

To distinguish the morphology of a flat and uneven surface, scanning tunneling microscope (STM) measurements were made on flat and uneven surfaces, as shown in Fig. S5. The uneven surface shows more steps (Fig. S5a). As for why the two spectra of areas 1 and 2 are so different, firstly, the well defined surface states measured on flat fresh surfaces are well understood. So the main question is why well defined surface states are not observed on the uneven fresh surfaces. As discussed in the main text, we believe that the suppression of surface states on uneven surfaces is related to the disorder of the surface. Specifically, STM measurements reveal numerous atomic-scale zigzag steps on the uneven surface (Fig. 2a), where each step hold many disordered structures caused by vacancy defects (Fig. 2b) and Nd atom clusters (Fig. 2c). The periodic potential of the surface crystal field fluctuates significantly along the step boundaries and within the disordered regions of vacancies

and clusters, disrupting the formation of a coherent long-range Bloch wave for the surface electronic state and, therefore, fully suppressing the surface states.

To visualize the evolution of surface morphology over time, at low temperatures, we recorded STM images at t=8 h and t=22 h after sample cleavage on the flat surface, as shown in Fig. S6. In STM measurements, locating an optimal sample area after cleavage is time-consuming, so by the time all preparations are complete, approximately 8 hours have typically passed since the sample was cleaved. It can be seen that many defects have appeared on the flat surface after 8 hours of sample cleavage. The defect area becomes larger with the increase of time (Fig. S6a-S6b). The depth of the defect is about 180 pm (Fig. S6d), which is consistent with the defect caused by the vacancy of an Nd atom. Given that ARPES measurements involve exposure to deep ultraviolet light, defect density is likely to increase further with exposure time. Supporting this, the electronic structure of the surface state shows a slight degradation 3 hours after sample cleave, when there is no UV light exposure (Fig. S7a). On the other hand, continuous UV irradiation during measurements significantly degrades the surface state structure (Fig. S7b). Therefore, the random control of disorder via UV assisted surface degradation under UHV conditions seems to be an inevitable trend[4, 5] in NdAlSi. It may be precisely due to the fact that the surface of NdAlSi is highly susceptible to degradation under ultraviolet irradiation. As a result, compared to previous Weyl semimetals, sufficient disorder can be introduced in NdAlSi, enabling the observation of a complete suppression of the Fermi arc on its topological surface and revealing the quantum phase transition driven by non-Anderson disorder.

5. Comparison of band structures between fresh surface and 10 hours after cleavage

To compare the electronic structure measured on a fresh flat surface and the flat surface after 10 hours of continuous measurement in more detail, we performed band structure measurements along Cut1-Cut4 (red lines in Fig. S8a), as shown in Fig. S8c-S8j. The band structure measured on the flat surface, after 10 hours of continuous measurement, look similar to that of the uneven fresh surface. Interestingly, after 10 hours of measurement, some of the bands appear sharper than when measured on the fresh surface (marked by green arrows in Fig. S8c-S8j). Therefore, we conclude that after 10 hours of continuous measurement, the surface of the sample has become disordered in a similar way as the fresh

uneven surface. This disorder seems to affect only the surface electronic structure as some of the bulk bands become sharper. A potential explanation is that the impurities are adsorbed at low temperatures, which would remove charge and weaken the bonds between Nd atoms and the surface. This increased mobility of Nd atoms results in a disordered surface with irregular clusters, similar to an uneven fresh surface.

6. Evolution of the band structures with time

To rule out the possibility that the disappearance of the surface Fermi arc (SFA) is the result not of surface degradation but rather from the SFA sharing the same dispersion as the bulk band, we conducted detailed time-dependent measurements of the SFA, as illustrated in Fig. S9. The Fermi arc is clearly visible for the first 7 hours, but after 10 hours of continuous measurement, it is no longer observed. Additionally, we performed energy distribution curve (EDC) analysis along the bulk band dispersion (see Fig. S9g). In the first 7 hours of continuous measurement, the EDC peak associated with the SFA was distinctly visible near the Fermi energy; however, after 10 hours of continuous measurement, this peak was no longer detectable. Furthermore, the EDC measured after 10 hours closely resembles that of a fresh uneven surface, with no discernible peak linked to the SFA, implying the complete disappearance of the Fermi arc.

7. Evolution of the electronic structures of NdAlSi under surface postassium evaporation

To address concerns regarding the uncontrollability of surface degradation, we carried out additional experiments in which disorder was deliberately introduced via surface potassium evaporation, allowing us to monitor the evolution of the Fermi arcs, as shown in Fig. S10. Interestingly, during potassium deposition on NdAlSi, the electronic structure did not undergo a uniform downward shift typically observed in other materials. Instead, its evolution closely tracked the increasing level of disorder introduced by the potassium. Additionally, we performed EDC analysis across the Fermi arc, as shown in Fig. S11. The EDC peak associated with the SFA was gradually suppressed and eventually vanished entirely with continued potassium evaporation.

Figure S12 shows the XPS measurements of NdAlSi on a flat fresh surface (red curves) and the surface after the last potassium evaporation (purple curves). In the XPS measure-

ments after potassium doping on the surface, the core levels corresponding to the K 3p orbital and a weak K 3s orbital can be clearly observed. Upon potassium adsorption on the NdAlSi surface, the intensity of all core-level peaks of NdAlSi decreases. Similar to the Si 2p orbital observed on the freshly cleaved rough surface, slight splitting or shifts also appear (Fig. S12). This slight splitting or shift is caused by the change in the chemical environment of Si induced by surface potassium evaporation.

8. Determination of the crystal structure of NdAlSi

The issue of Al/Si site disorder in ReAlSi compounds has been debated for a long time. This is primarily because the noncentrosymmetric space group $I4_1/md$ (no. 109) and centrosymmetric space group $I4_1/amd$ (no. 141) are indistinguishable by X-ray diffraction for NdAlSi, and their contrast in neutron diffraction is small. Nevertheless, we identified three key pieces of evidence indicating that NdAlSi is a non-centrosymmetric crystal rather than a centrosymmetric one.

I. Recently, second-harmonic generation (SHG) measurements have been reported as an effective method for distinguishing between these structures in the ongoing debate[1]. The SHG measurements points to the noncentrosymmetric space group $I4_1md$ (no. 109) and is inconsistent with the centrosymmetric space group $I4_1/amd$ (no. 141).

II. As shown in Fig. 1c–1d of the manuscript, the measured Fermi surface on the Nd-terminated surface aligns well with the corresponding DFT calculations based on the non-centrosymmetric space group $I4_1/md$ (no. 109).

III. One of the main differences between noncentrosymmetric crystals and centrosymmetric crystals is whether the elements of the two terminating surfaces after crystal cleavage are antisymmetric or symmetric. Taking NdAlSi as an example, the easy cleavage plane of NdAlSi along the direction (001) is the Nd-Al plane (Cut 1 in Fig. S13a). If NdAlSi is non-centrosymmetric, fixing the front and back sides of the single crystal (e.g., with Nd atoms on the top surface and Al atoms on the bottom) ensures that after any number of cleaves, the lower surface of the upper crystal half will consistently have an Al termination, while the upper surface of the lower half will have an Nd termination. In contrast, if NdAlSi were centrosymmetric (with Al and Si atoms randomly occupying each other's Wyckoff positions, as shown in Fig. S13b), the probability of cleavage along Cut 1 and Cut 2 would be equal. Consequently, the upper and lower surfaces after cleavage would be indistin-

guishable, and Nd and Al terminations would appear in different regions across the same cleavage surface. Through a large number of repeated experiments (Fig. S14), the results support that NdAlSi is a noncentrosymmetric crystal. That is, once the NdAlSi crystal is set in a fixed orientation, each cleavage consistently produces asymmetric upper and lower planes, with the element termination on each cleavage surface remaining unchanged. There is only one surface state of the same flat cleavage. This conclusion is also consistent with the STM measurements that the upper and lower surface after cleavage exhibit totally different atomic morphology (Fig. S15). Some atomic-level defects are present in the STM measurements of the Al atomic terminal surface (Fig. S15b), and we cannot entirely rule out the possibility that these defects may be linked to Al/Si site disorder, even so, this disorder is localized on a small scale. Some detailed measurement results are also described in another manuscript[3].

9. Determination of surface state features

To clarify the surface-state origin of the diamond-shaped Fermi surface observed in Fig. 3i–3l, we performed a more detailed analysis. Fig. S16a-S16d showns the photon energy dependent band structures measured on fresh surface of flat sample with photon energies of 30 eV (Fig. S16a), 41 eV (Fig. S16b), 49 eV (Fig. S16c) and 53 eV (Fig. S16d) along Cut1 in Fig. 3i. The red lines in Fig. S16a-S16d represent the same set of data and consistently trace the corresponding band features in Fig. S16a-S16d. Furthermore, momentum distribution curve (MDC) analysis (Fig. S16e) shows that the band features corresponding to the peaks marked by dashed lines exhibit negligible photon energy dependence, indicating that they originate from a surface state.

10. Definition of diffusive metal

A diffusive metal is a type of metallic system where electron transport is dominated by scattering, primarily from disorder, phonons, or impurities, rather than by coherent or ballistic motion. In conventional diffusive metals that preserve Fermi liquid behavior, quasiparticles are well-defined, but their motion becomes randomized due to frequent scattering, leading to diffusive transport. The resistance-temperature R(T) behavior in such systems is determined by the dominant scattering mechanism.

1. For impurity scattering, R(T) remains approximately constant at low temperatures;

2. Under electron–phonon scattering, R(T)∼T^5 at low temperatures and becomes linear R(T)∼T at higher temperatures;

3. For electron–electron interactions, typical of a Fermi liquid, R(T)∼T^2.

In contrast, in bad metals or strongly correlated systems near a Mott transition, quasiparticles become poorly defined or entirely absent, reflecting a breakdown of conventional Fermi liquid theory. Due to the presence of numerous impurities or defects on the disordered surface, when the topological surface Fermi arc is completely suppressed, the electron transport behavior in the quasi-two-dimensional region near the surface no longer exhibits topological characteristics. Instead, the electron transport is dominated by disorder scattering and manifests as diffusive metal behavior.

---

[1] J. Gaudet *et al.*, Weyl-mediated helical magnetism in NdAlSi. Nat. Mater. **20**, 1650 (2021).

[2] C. Li *et al.*, Emergence of Weyl fermions by ferrimagnetism in a noncentrosymmetric magnetic Weyl semimetal. Nat. Commun. **14**, 7185 (2023).

[3] C. Li *et al.*, Non-Hermitian Boundary in a Surface Selective Reconstructed Magnetic Weyl Semimetal. Adv. Mater. 2419559 (2025).

[4] R. Jiang *et al.*, Reversible tuning of the surface state in a pseudobinary $Bi_2(Te\text{-}Se)_3$ topological insulator. Phys. Rev. B **86**, 085112 (2012).

[5] Y. Wang *et al.*, Giant and reversible electronic structure evolution in a magnetic topological material $EuCd_2As_2$. Phys. Rev. B **106**, 085134 (2022).

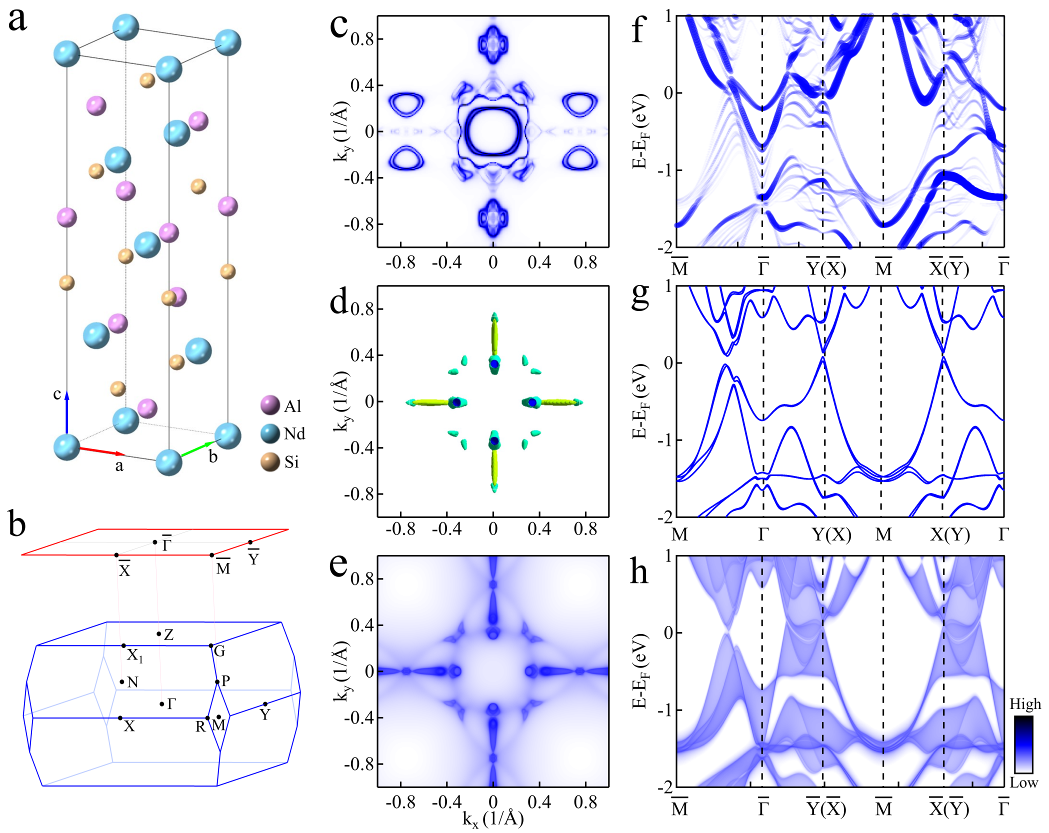

FIG. S1. **Crystal structure and calculated electronic structure of NdAlSi.** (a) The crystal structure of NdAlSi with the space group $I4_1md$ (no. 109). (b) The 3D BZ of the original unit cell of NdAlSi, and the corresponding two-dimensional BZ projected on the (001) plane (red lines) in the pristine phase in (a). (c) Surface projected DFT calculated Fermi surface on the terminal surface of Nd atoms cleavage at the Al-Nd layer. (d) The DFT calculated 3D bulk Fermi surface of NdAlSi. (e) The DFT calculated bulk Fermi surface of NdAlSi which integrate all of the BZ along k_z direction. (f) The surface projected DFT calculated band dispersion along $\overline{M}-\overline{\Gamma}-\overline{Y}-\overline{M}-\overline{X}-\overline{\Gamma}$ directions on the terminal surface of Nd atoms cleavage at the Al-Nd layer. (g) Calculated band structures of NdAlSi along high-symmetry directions across the BZ. (h) The DFT calculated bulk band structure of NdAlSi which integrate all of the BZ along k_z direction.

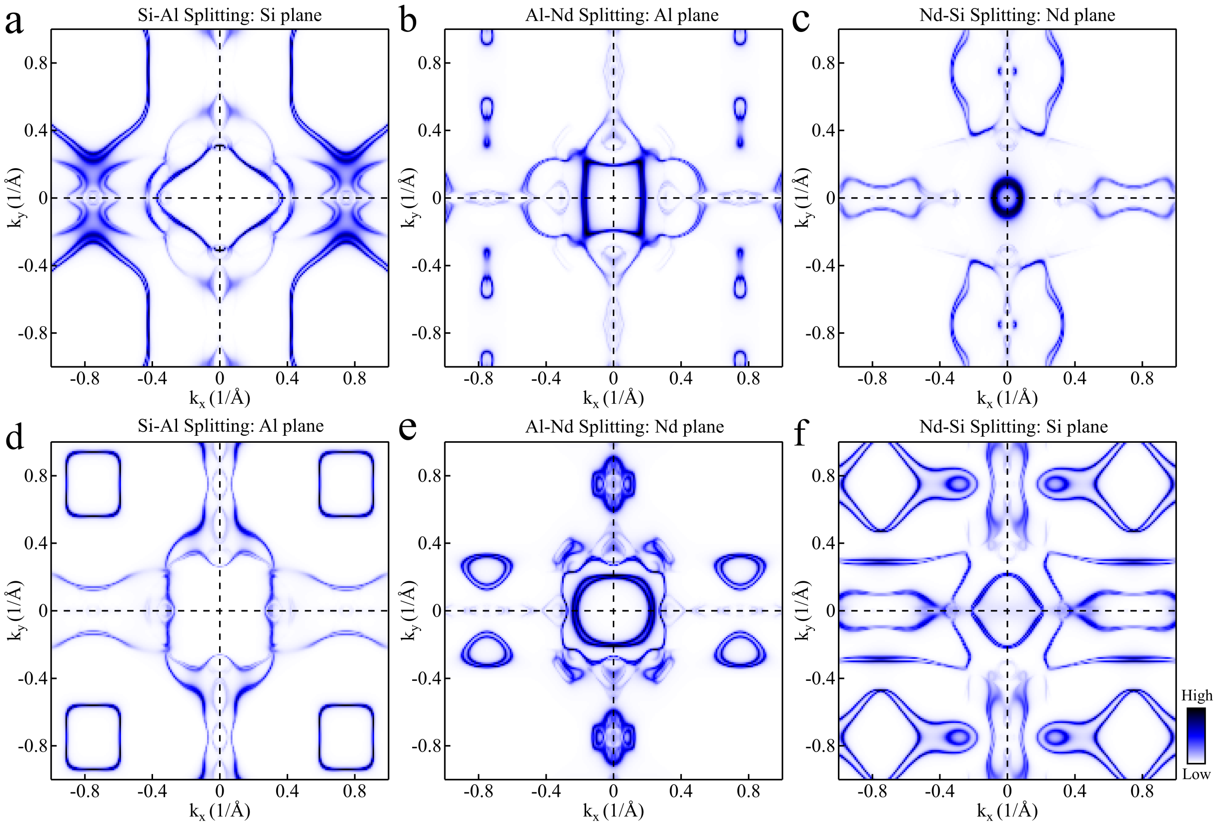

FIG. S2. **Surface projected DFT Fermi surfaces of NdAlSi.** (a-f) The surface projected DFT calculated Fermi surfaces on the Si terminated surface (a) and Al terminated surface (d) cleaved at the Si-Al layer, Al (b) and Nd (e) terminated surfaces at the Al-Nd layer, Nd (e) and Si (f) terminated surfaces at the Nd-Si layer.

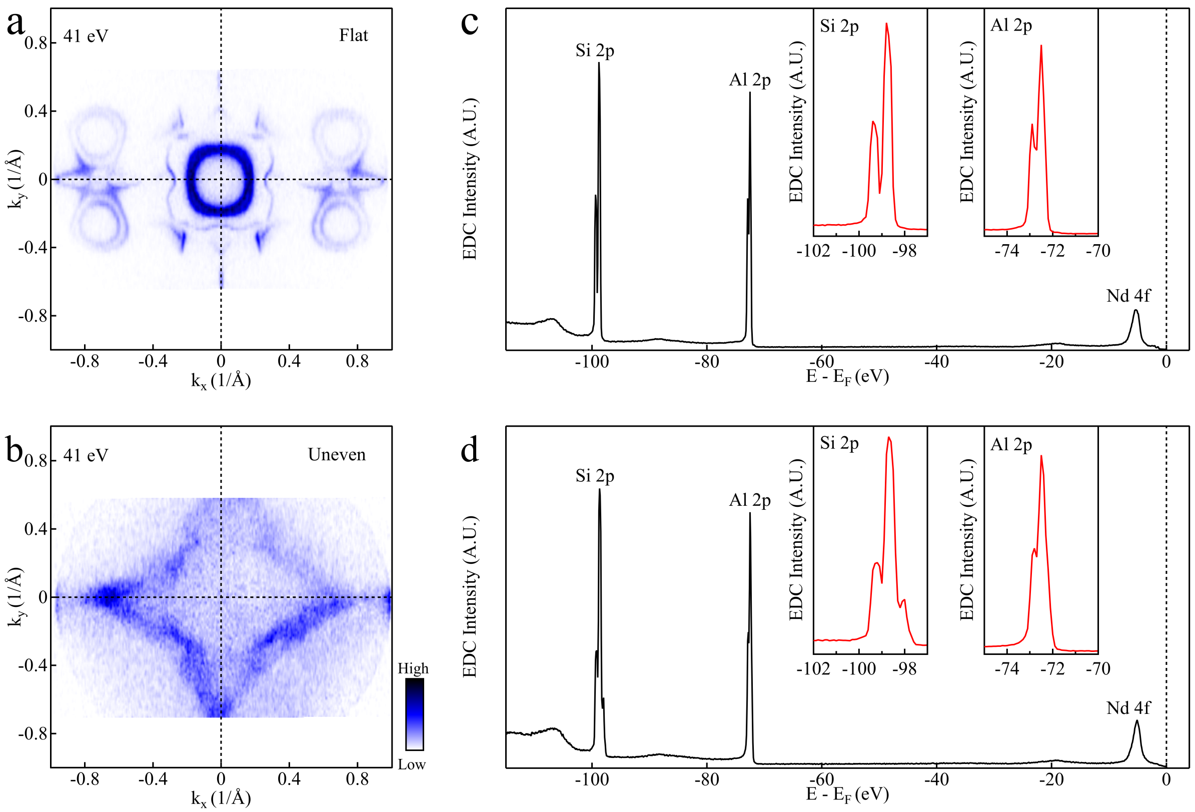

FIG. S3. (a-b) The Fermi surface of NdAlSi measured with photon energy of 41 eV on flat (a) and uneven (b) surface. (c-d) The corresponding XPS measurements on flat (c) and uneven (d) surfaces. The inset presents an enlarged view of the Si and Al 2p orbitals.

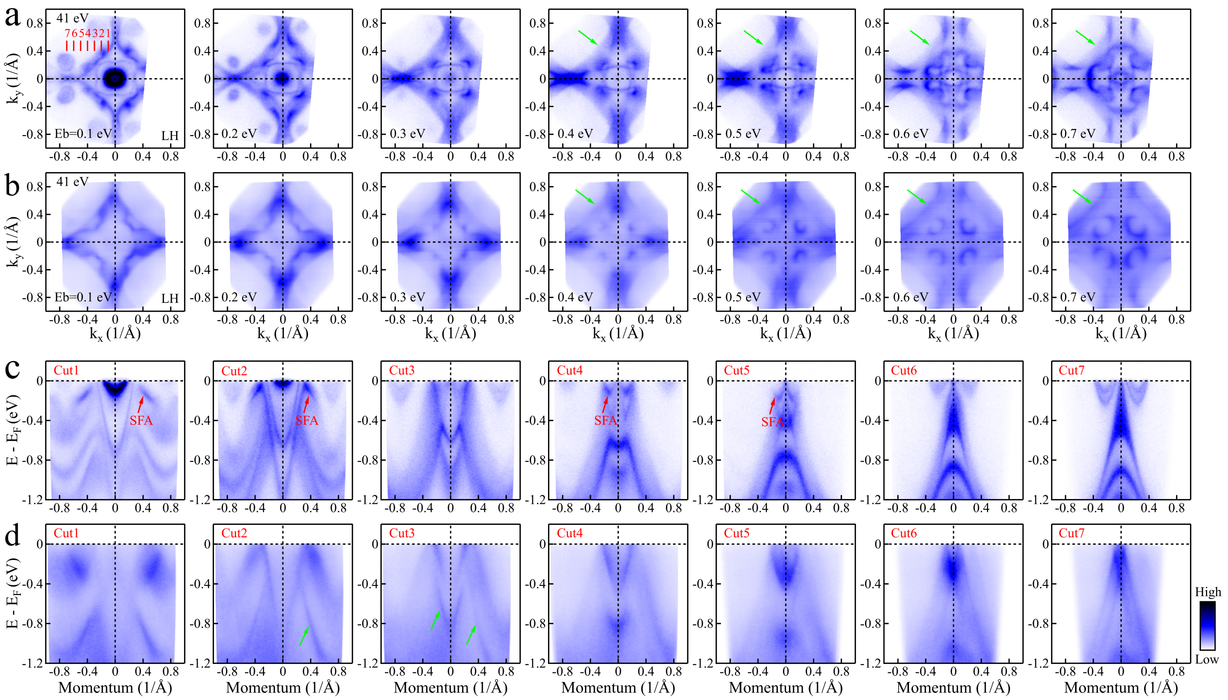

FIG. S4. **Comparison of constant energy contours and band structure of flat and uneven surfaces.** (a) The constant energy contous of NdAlSi measured in the area 1 (red circle in Fig. 2a) of sample with photon energy of 41 eV under LH polarization. (b) The constant energy contous of NdAlSi measured in the area 2 (orange circle in Fig. 2k) of sample with photon energy of 41 eV under LH polarization. (c-b) The band structure measured in the area 1 (c) and area 2 (d) of sample along Cut1-Cut7 directions with photon energy of 41 eV under LH polarization. The cut positions are marked by red lines in (a). The SFAs are marked by red arrows in (c).

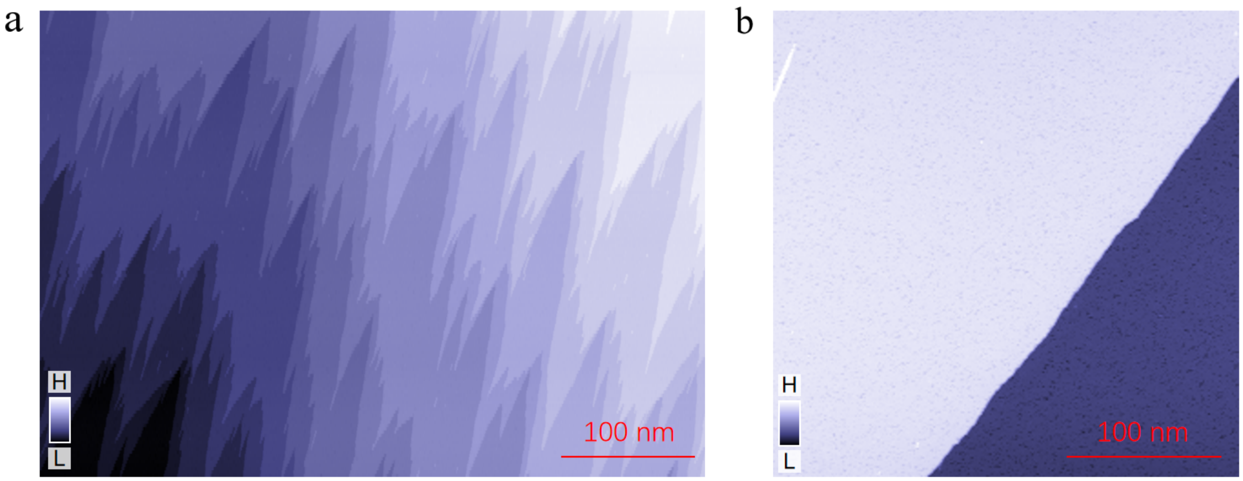

FIG. S5. STM images of NdAlSi measured on uneven (a) and flat (b) Nd atom terminated surface cleavage at the Al-Nd layer. Scan conditions: (a) 1 V, 0.1 nA, (b) 0.5 V, 0.5 nA.

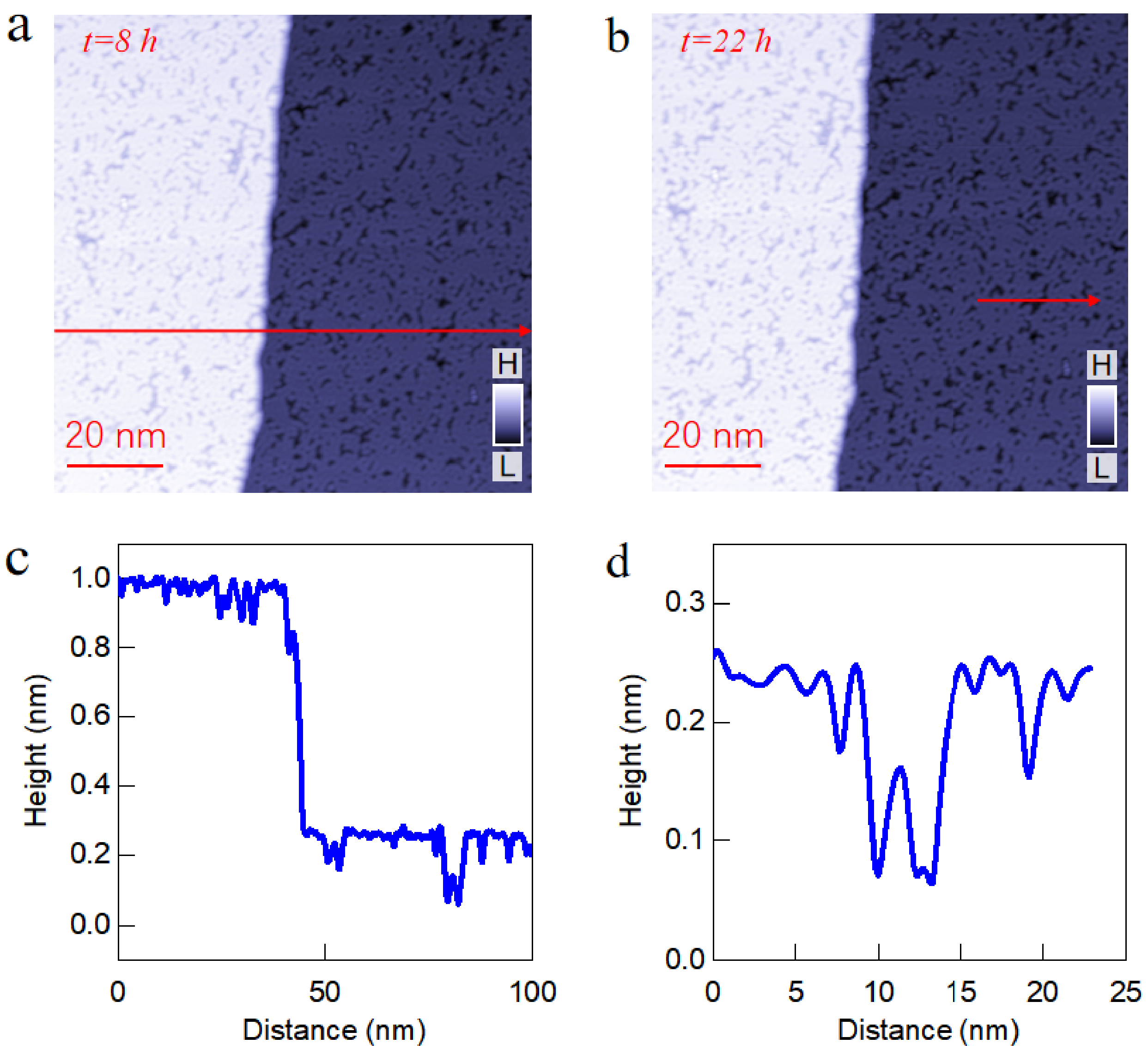

FIG. S6. STM images of NdAlSi measured at 4.7 K on flat Nd atom terminated surface at 8 h (a) and 22 h (b) after sample cleavage. Line profile along the red line in (a) and (b) are shows in (c) and (d). Scan conditions: (a-b) 0.1 V, 0.01 nA.

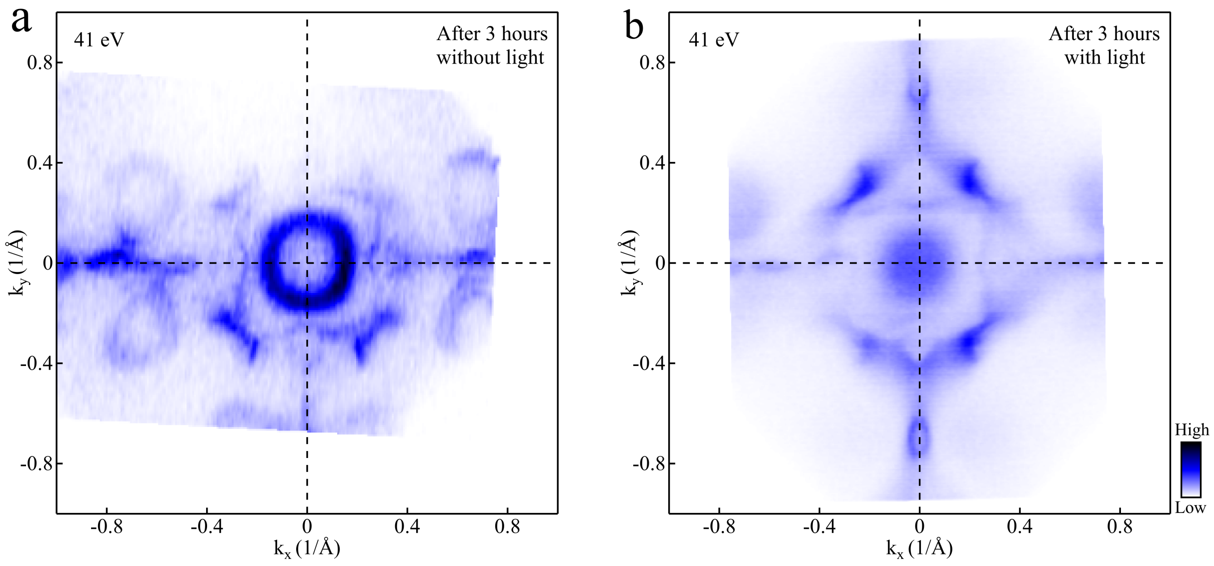

FIG. S7. The Fermi surface of NdAlSi measured with photon energy of 41 eV on a flat area of the sample 3 hours after cleavage without (a) and with (b) continuous ultraviolet irradiation.

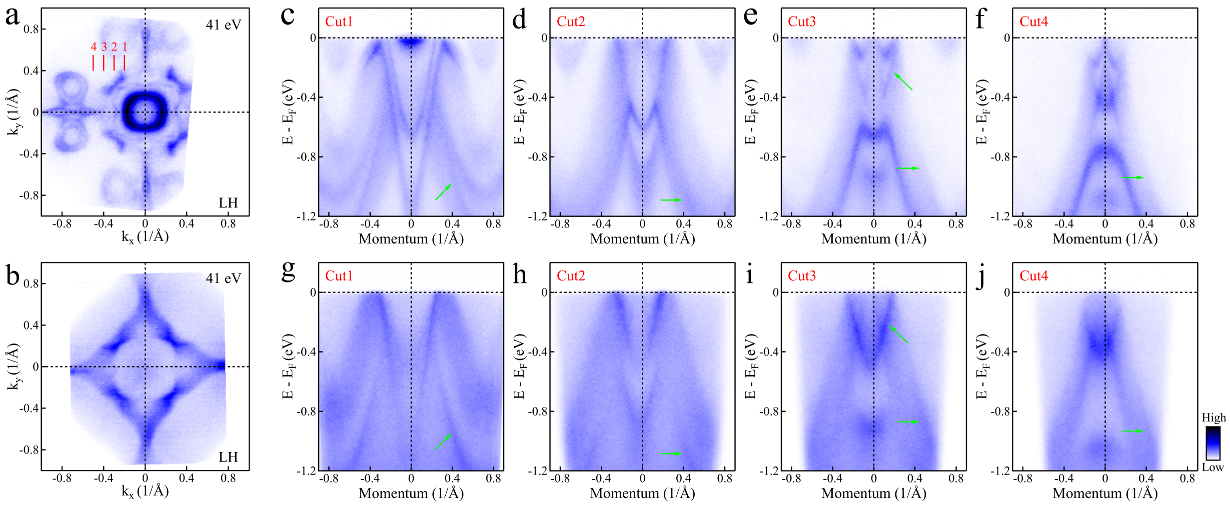

FIG. S8. **Comparison of band structure measured on a fresh surface and the surface after 10 hours of continuous measurement.** (a) The constant energy contours of NdAlSi measured in the area 1 (red circle in Fig. 2a) of the fresh surface (a) and the surface after 10 hours of continuous measurement (b) with photon energy of 41 eV under LH polarization. (c-f) The band structures measured in the area 1 of fresh surface along Cut1-Cut4 directions with photon energy of 41 eV under LH polarization. (g-j) Similar measurements as (c-f) but recorded after 10 hours of continuous measurement.

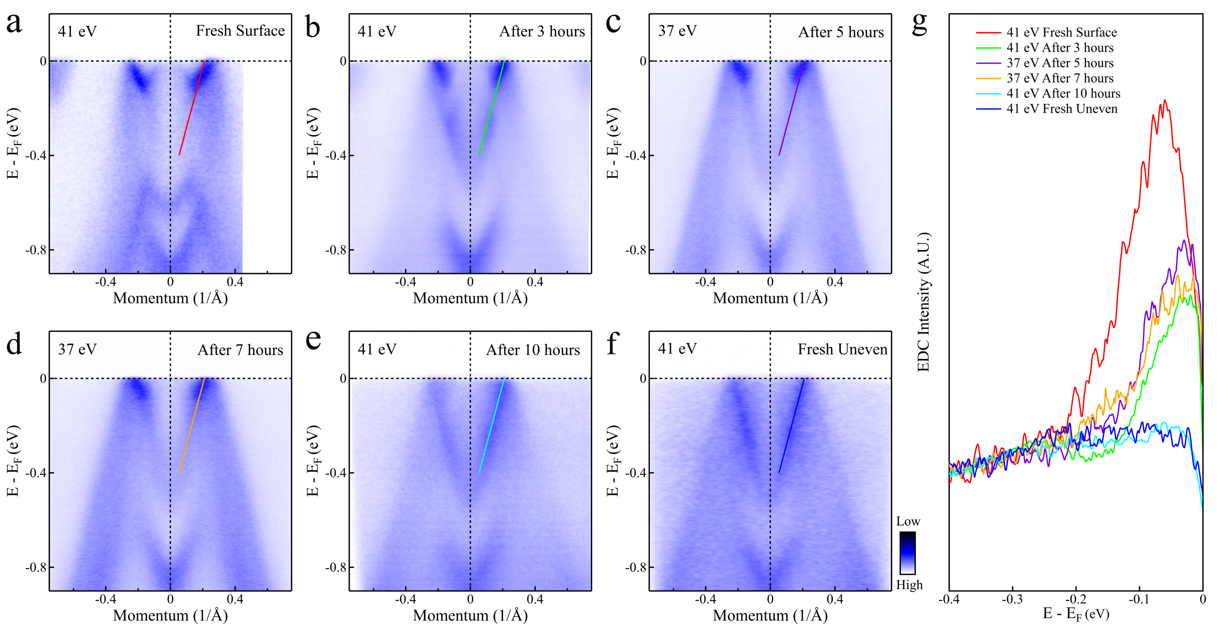

FIG. S9. **Evolution of the band structures with time.** (a-e) The band structure crossing the Fermi arc measured with photon energies of 41 eV (a, b, e) and 37 eV (c-d) on flat area of the fresh surface (a), the surface after 3 hours of continuous measurement (b), the surface after 5 hours of continuous measurement (c), the surface after 7 hours of continuous measurement (d) and the surface after 10 hours of continuous measurement (e). (f) The band structure along the same momentum cut as (a-e) but measured on a fresh uneven surface. (g) The energy distribution curves (EDCs) extracted along the sloping line in panels (a-f). All the EDCs are normalized at energy range of -0.4 eV to -0.3 eV.

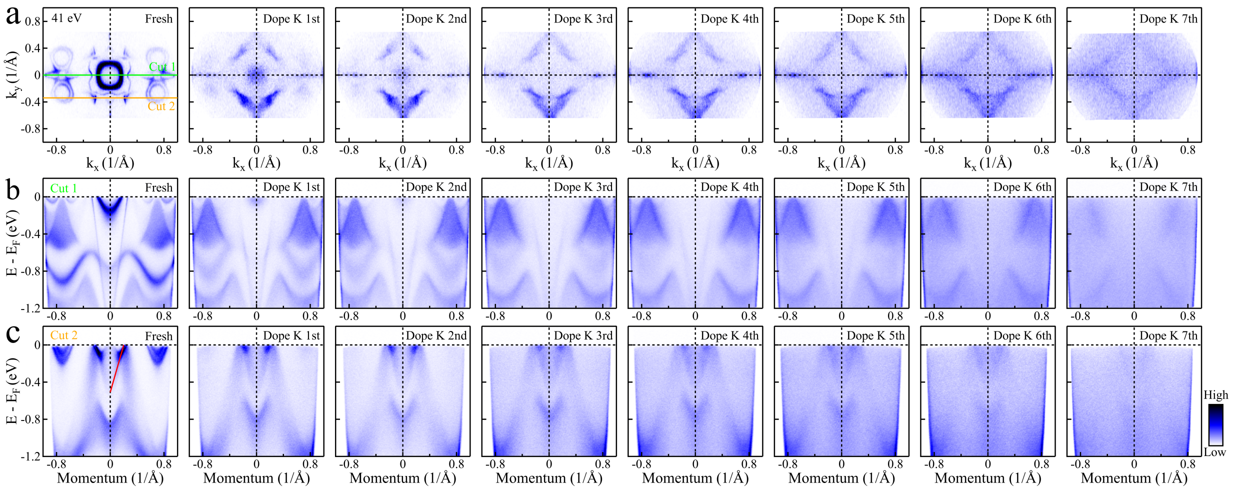

FIG. S10. **Evolution of the electronic structures of NdAlSi under surface potassium evaporation.** (a) Evolution of the Fermi surface as a function of potassium deposition. (b-c) Corresponding evolution of the band dispersion along cut 1 (b) and cut 2 (c) with increasing potassium evaporation. Each Fermi surface measurement was completed within 10 minutes. The conditions for each potassium evaporation are shown in the table in Fig. S11.

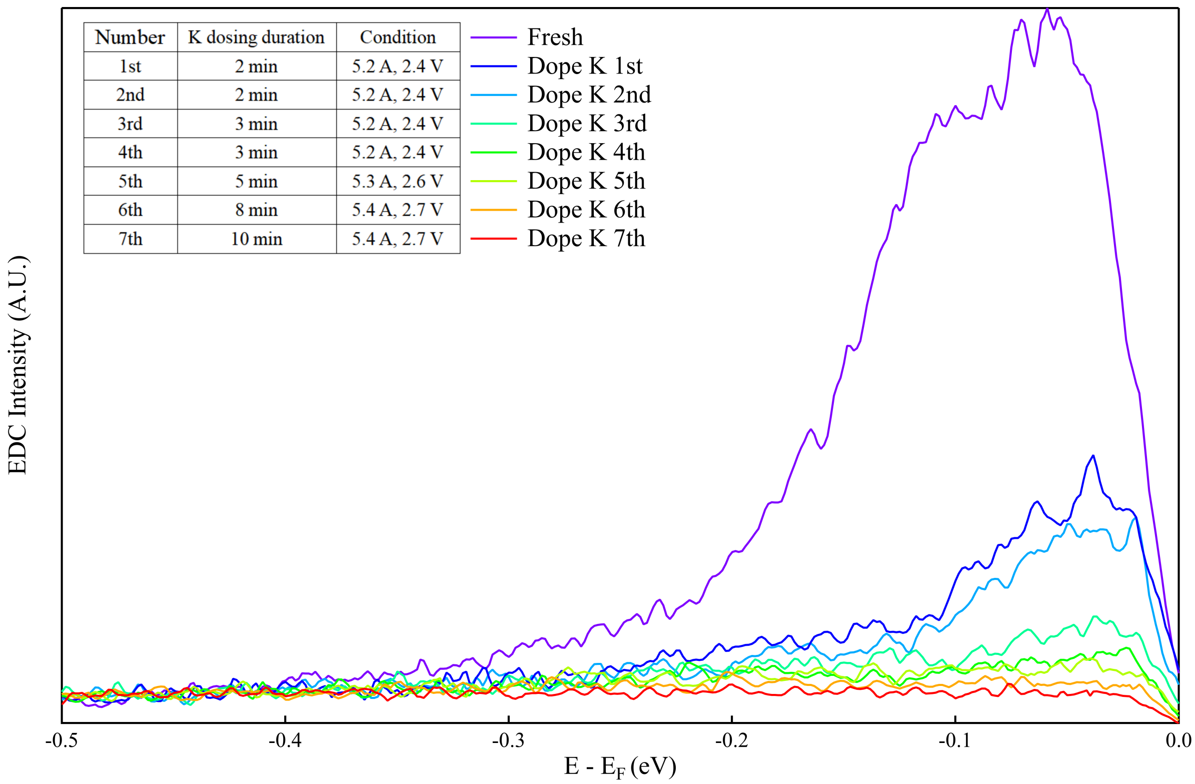

FIG. S11. Evolution of the EDCs extracted along the sloping line in Fig. S10c with increasing potassium evaporation. All the EDCs are normalized at energy range of -0.5 eV to -0.4 eV.

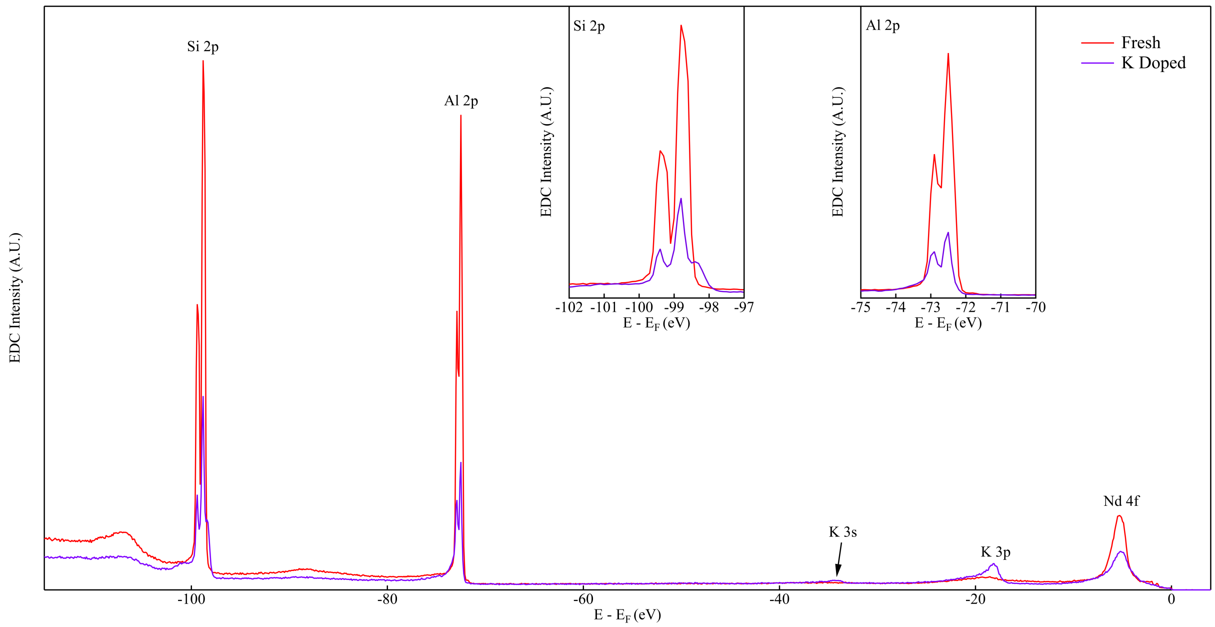

FIG. S12. XPS measurements on a flat fresh surface and the surface after the last potassium evaporation. The inset presents an enlarged view of the Si and Al 2p orbitals.

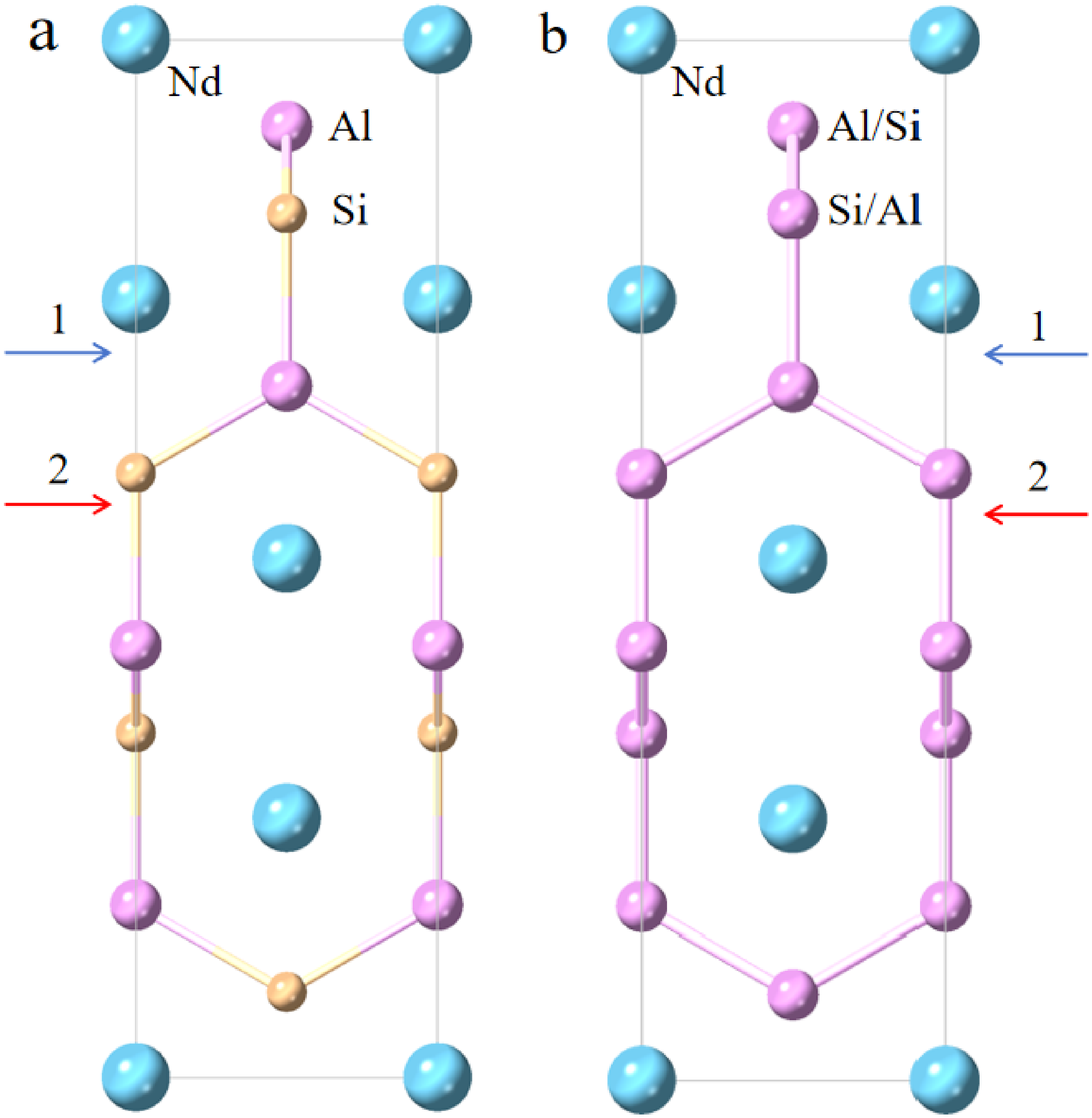

FIG. S13. **NdAlSi crystal structure diagrams of two different space groups.** (a) and (b) show the NdAlSi crystal structure with the cnoncentrosymmetric space group I41/md (no. 109) and the centrosymmetric space group I41/amd (no. 141).

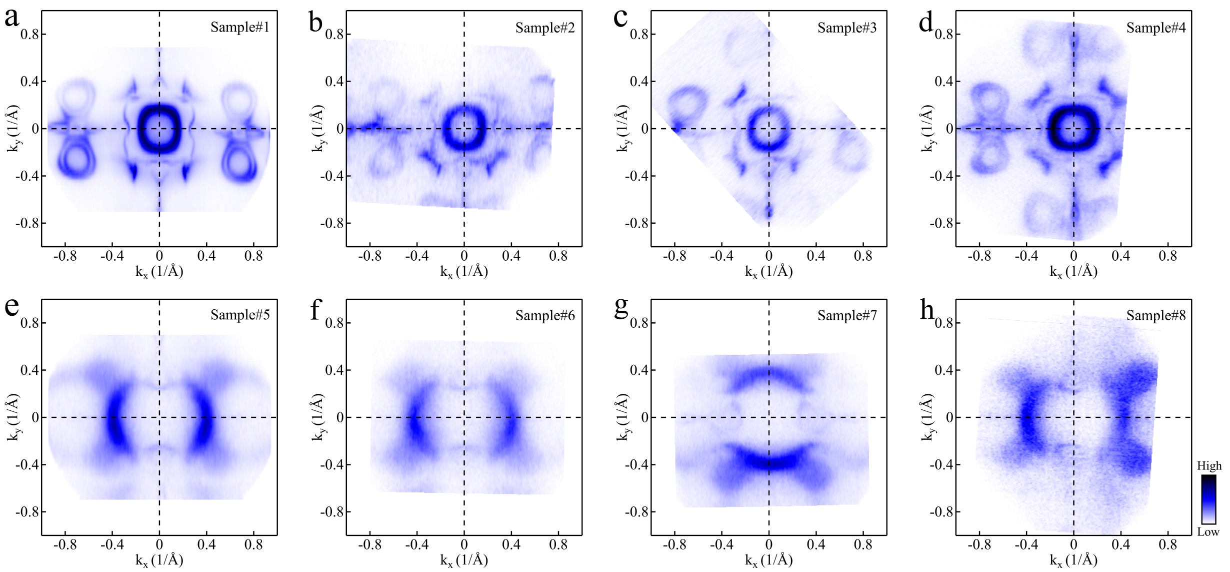

FIG. S14. **Fermi surface of NdAlSi measured from 8 different samples with photon energy of 41 eV.** The measured Fermi surfaces of (a-d) correspond to Nd atom terminated surface cleavage at the Al-Nd layer, and the measured Fermi surfaces of (e-h) correspond to Al atom terminated surface cleavage at the Al-Nd layer.

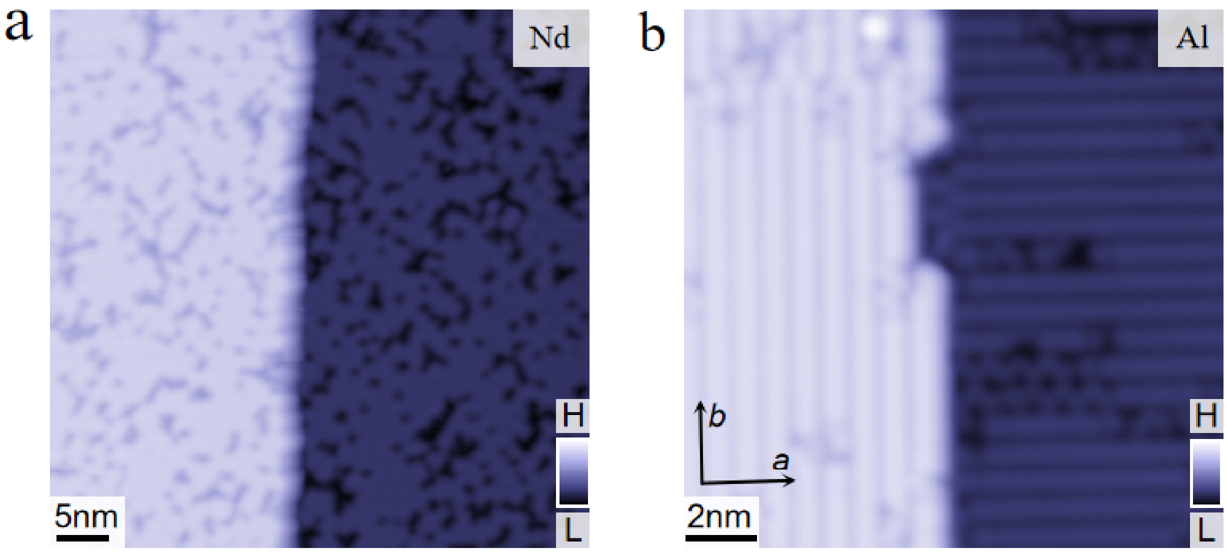

FIG. S15. STM images of NdAlSi measured on Nd atom terminated surface (a) and on Al atom terminated surface (b) cleavage at the Al-Nd layer. Scan conditions: (a) -0.5 V, 0.5 nA, (b) -1.5 V, 0.5 nA.

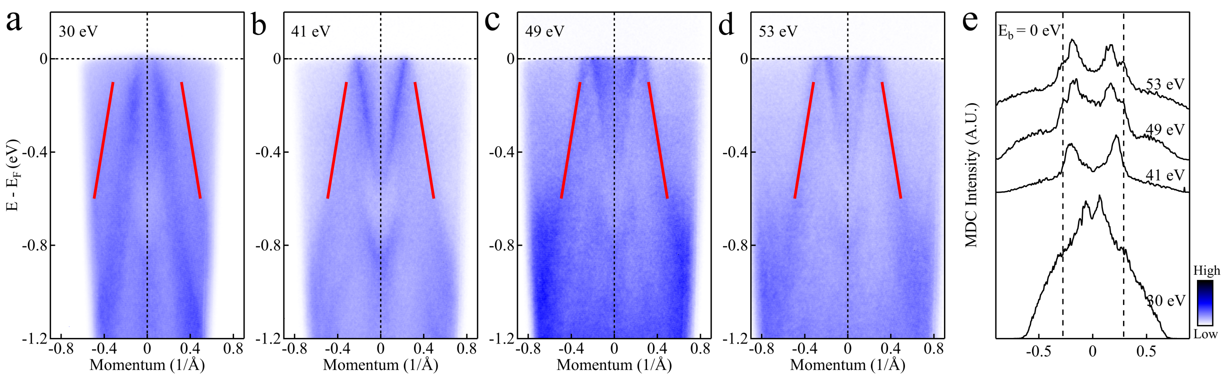

FIG. S16. (a-d) Photon energy dependent band structures measured on fresh surface of flat sample with photon energies of 30 eV (a), 41 eV (b), 49 eV (c) and 53 eV (d) along Cut1 in Fig. 3i. (e) The momentum distribution curves (MDCs) extracted from (a-d) at Fermi energy. The red curves in (a-d) represent the same set of data, and the dashed lines in (e) indicate the momentum positions of the peaks in the MDC corresponding to the surface states.
